# Strategy Development and Feedback Processing During Complex Category Learning

**DOI:** 10.3389/fpsyg.2021.672330

**Published:** 2021-11-10

**Authors:** Victoria Tilton-Bolowsky, Sofia Vallila-Rohter, Yael Arbel

**Affiliations:** MGH Institute of Health Professions, Boston, MA, United States

**Keywords:** strategy development, category learning, feedback processing, feedback related negativity, probabilistic learning

## Abstract

In this study, 38 young adults participated in a probabilistic A/B prototype category learning task under observational and feedback-based conditions. The study compared learning success (testing accuracy) and strategy use (multi-cue vs. single feature vs. random pattern) between training conditions. The feedback-related negativity (FRN) and P3a event related potentials were measured to explore the relationships between feedback processing and strategy use under a probabilistic paradigm. A greater number of participants were found to utilize an optimal, multi-cue strategy following feedback-based training than observational training, adding to the body of research suggesting that feedback can influence learning approach. There was a significant interaction between training phase and strategy on FRN amplitude. Specifically, participants who used a strategy in which category membership was determined by a single feature (single feature strategy) exhibited a significant decrease in FRN amplitude from early training to late training, perhaps due to reduced utilization of feedback or reduced prediction error. There were no significant main or interaction effects between valence, training phase, or strategy on P3a amplitude. Findings are consistent with prior research suggesting that learners vary in their approach to learning and that training method influences learning. Findings also suggest that measures of feedback processing during probabilistic category learning may reflect changes in feedback utilization and may further illuminate differences among individual learners.

## Introduction

The human ability to categorize is a fundamental behavior that supports recognition and underlies concept formation (Zentall et al., 2002; Ashby and O’Brien, 2005; Waxman and Gelman, 2009). Categorization helps people recognize that a bird seen for the first time, for example, is a bird, even if it is not like any bird the person has ever encountered. Categorization can influence decisions based on category membership (e.g., poisonous vs. edible berry) or support recognition (e.g., help listeners distinguish familiar and unfamiliar voices). While category knowledge is often acquired unconsciously through exposure and experience, category knowledge can also be acquired in more structured ways. Research suggests that the process and degree to which a category is learned can depend upon external factors related to category structure and/or method of instruction, and also on factors about the learner, such as cognition (Knowlton et al., 1994; Ashby et al., 2002; Gluck et al., 2002; Ashby and O’Brien, 2005; Bozoki et al., 2006; Ashby and Maddox, 2011; Karcher et al., 2019; Feldman, 2021). Particularly in instances of acquired brain damage or disease where concepts must be re-formed, re-learned, or reinforced, understanding the interaction between external and individual factors that affect the learning process can serve to benefit the design of optimal learning conditions (Knowlton and Squire, 1993; Bozoki et al., 2006; Karcher et al., 2019). The present study focused on complex multi-dimensional category learning and examined how an external factor, the presence or absence of feedback, influenced learning success and strategy development. The present study further examined how individual differences in feedback processing may relate to learning strategy and success during feedback-based category learning.

Many of the categories and concepts we encounter as humans are complex, with category membership determined by multiple dimensions and/or complex rules that are not easily verbalized (Zentall et al., 2002). Within a research context, studies that examine category learning of complex categories utilize paradigms such as: information integration tasks, in which a category boundary is dependent upon multiple dimensions (e.g., the thickness and orientation of lines within a geometric shape); prototype-distortion tasks, in which a prototypical item is selected and within-category items contain minor differences or distortions from the prototype; and probabilistic tasks, in which an outcome or association is governed by a probabilistic association of a certain feature or combination of features with an outcome (e.g., cards with diamonds predicting rain on 80% of trials; Knowlton and Squire, 1993; Ashby and O’Brien, 2005; Ashby and Maddox, 2011). With probabilistic category tasks of this kind, information from a single trial is not reliable or sufficient to give the learner a full representation of the category boundary. Rather, for optimal learning, information must be accrued across many trials (Knowlton et al., 1994).

Research proposes the existence of two systems that are differentially engaged to support category learning: rule-based and implicit systems. Rule-based systems are thought to use declarative, explicit processes of hypothesis generation and testing, and draw upon working memory and executive resources to reason through possible category rules and evaluate outcomes (Ashby and O’Brien, 2005; Ashby and Maddox, 2011). Rule-based systems are well-suited for learning categories with few dimensions. Alternatively, implicit systems are thought to depend upon dopamine-mediated reinforcement learning in the basal ganglia, which supports the nondeclarative and gradual learning of categories that occurs below one’s conscious awareness (Ashby and Maddox, 2011). Implicit systems are particularly well-suited for processing and learning complex patterns. While certain category structures may be best learned *via* one system vs. the other, and certain tasks may be described as either rule-based or implicit, learners differ in their approach to learning. For example, a learner may use a rule-based approach even for a task that contains too many dimensions to integrate effectively *via* verbalization, hypothesis testing, and updating (Gluck et al., 2002; Vallila-Rohter and Kiran, 2015).

Gluck et al. (2002) investigated the strategies that individuals used while participating in the weather prediction task (WPT), a probabilistic classification task in which individuals are instructed to predict outcomes of sun or rain based on combinations of cue cards displaying geometric shapes (Knowlton et al., 1994). Challenging the assumption that the probabilistic nature of the task leads learners to gradually accrue knowledge across multiple dimensions (Gluck and Bower, 1988), the authors identified three different approaches/strategies employed by their participants: (1) A multi-cue strategy in which participants based responses on combinations of cues (i.e., combinations of geometric shapes), (2) a one-cue strategy in which participants-based responses on the presence or absence of a single cue within the combination, or (3) a singleton strategy in which participants focused learning only when a single cue was presented and guessed on trials where two or more were presented (Gluck et al., 2002). The authors also noted that some participants demonstrated a shift in strategy from the early to the late stages of the task, and those who were using a multi-cue strategy during the later phases of learning made higher proportions of optimal responses and achieved higher accuracy than the singleton strategy users (Gluck et al., 2002). Thus, while the WPT may be best learned *via* a multi-cue strategy, strategy analyses such as these suggest that category structure alone does not determine the system or approach utilized to learn a category. Rather, individuals vary in the strategies they use to learn, which in turn, influences success. This study innovatively used behavioral analyses to specifically examine learner *strategies and approaches* to learning, and looked beyond purely learning outcomes. This work demonstrated the value of such strategy analyses and laid a foundation for future work evaluating learner strategies under a variety of learning tasks, with the goal of evaluating whether similar patterns arise. For example, studies have applied strategy analyses to data from reinforcement learning (Schulz et al., 2018), experience-based decision making (Choung et al., 2017), as well as category learning in children (Visser and Raijmakers, 2012; Rabi and Minda, 2014), aging individuals (Maddox et al., 2010), and clinical populations such as aphasia, amnesia, and Parkinson’s Disease (Shohamy et al., 2004; Meeter et al., 2006; Vallila-Rohter and Kiran, 2015). More research, however, is needed to understand the variety and consistency of strategies utilized when learners approach complex learning tasks and how task manipulations and learner factors influence strategy development.

Manipulating a category learning task’s conditions has been found to influence neural activation (Poldrack et al., 2001). Poldrack et al. (2001) collected functional magnetic resonance imaging data on learners who completed the WPT under observational and feedback-based conditions. The authors found that the medial temporal lobe showed activation in observational learning conditions, while the caudate nucleus of the basal ganglia showed activation during feedback-based learning (Poldrack et al., 2001). Manipulating a category learning task’s conditions has also been found to influence the behavioral strategies that participants develop and employ (Ashby et al., 2002; Vallila-Rohter and Kiran, 2015; Bellebaum et al., 2016). Of particular relevance to the current work, research has found that manipulating feedback and response method can influence learning processes and success (Ashby and Maddox, 2011). Ashby et al. (2002) examined learning processes and success using a category learning task (Ashby and Gott, 1988) under two training conditions: feedback training and observational training, and across two category structures: a rule-base category structure and an information-integration category structure. The authors found that for the information-integration category, where the category boundary depended upon information integrated across multiple dimensions, learning outcomes were better following training in which the correct stimulus classification was shown *after* the stimulus itself (the feedback condition), relative to training in which the correct stimulus classification was shown *before* the stimulus was presented (the observational condition; Ashby et al., 2002). These findings suggest that task condition can influence learning success. Further, providing learners with category information during the learning process and *after* they are exposed to exemplars may be important for learning: the training condition in which the correct classification was shown *after* the stimulus was thought to support the development of optimal strategies, as more participants progressed from a simple, unidimensional rule to an optimal, multidimensional rule under this condition. Strategy analyses offer a window into the process of learning, but further research is needed to confirm whether these strategies consistently correspond to the recruitment of distinct neural mechanisms associated with declarative (explicit) and nondeclarative (implicit) learning. While single cue and multi-cue strategies have been described as declarative and nondeclarative, respectively – as noted by Gluck et al. (2002), it is not because a strategy such as a single cue strategy can be easily verbalized and consciously recalled that it is always applied declaratively by learners.

Learning accuracy and strategy analyses provide information specific to learning *outcomes*, which is undeniably valuable. However, these measures do not provide information about what occurs *during* the learning process and, relatedly, what occurs during the learning process as a result of feedback. Much remains unknown about how individuals process feedback and if/how the provision of feedback impacts strategy development.

Event-related potentials (ERP) extracted from electroencephalography (EEG) by means of signal averaging enables researchers to measure how individuals process feedback and can provide insights into the mechanisms associated with particular strategies during learning. ERPs are temporally-sensitive patterns of changes in voltage that are evoked by discrete events, and are assumed to represent specific cognitive processes and reflect brain activity (Kutas et al., 2006). The feedback-related negativity (FRN) ERP was first described by Miltner et al. (1997) who employed a time-estimation task guided by feedback. The FRN is a fronto-central negativity that peaks between 200 and 300ms (Miltner et al., 1997; Holroyd and Coles, 2002; Hauser et al., 2014) following external feedback, and is larger following negative feedback than positive feedback (Holroyd and Coles, 2002; Ernst and Steinhauser, 2012). The FRN is thought to be elicited by external error feedback (Nieuwenhuis et al., 2004; Eppinger et al., 2009) and to reflect reward prediction error processing (i.e., when the anticipated outcome and actual outcome differ; Holroyd and Coles, 2002; Hauser et al., 2014; Burnside et al., 2019). The FRN has been found to decrease with learning over time, which may indicate either that the prediction error becomes smaller with learning (Burnside et al., 2019), or that feedback becomes less useful over the course of learning (Holroyd and Coles, 2002; Arbel and Wu, 2016). The FRN is thought to be generated within the mesencephalic dopamine system, often described as important for implicit learning, though theories of reinforcement learning relate the FRN to more explicit processes of hypothesis testing (see Luft, 2014). The FRN has been discussed more recently as reflecting a reward-related positivity, or a positivity that is absent or suppressed following negative performance feedback, in turn producing a negativity (Holroyd et al., 2008; Proudfit, 2015). Overall, the discovery of the FRN has allowed researchers to measure individuals’ processing of feedback, and thus, examine the relationship between feedback processing and learning outcomes. The processing of feedback, especially corrective feedback, is integral to successful learning.

There is another ERP that follows the FRN and is also related to feedback processing and learning, termed the “P3a” by Butterfield and Mangels (2003). The P3a is a fronto-central positivity that peaks between 200 and 400ms following the provision of negative feedback and is thought to reflect processing of feedback-related and reward-related variables such a feedback valence and probability (Butterfield and Mangels, 2003; Arbel et al., 2013; Rustemeier et al., 2013). The P3a, sometimes described as a subcomponent of the P300, has been found to be associated with learning outcomes (Arbel et al., 2013) and is thought to be the product of attentional orientation with the goal of detecting and evaluating unexpected events and facilitating performance error correction for subsequent action (Friedman et al., 2001; Butterfield and Mangels, 2003). The evaluation of unexpected events (e.g., negative feedback over the course of learning) to correct future performance is also integral to successful learning.

Few studies have examined how feedback processing relates to strategy development. Rustemeier et al. (2013) set out to do so using a modified version of the WPT and evaluating measures of feedback processing as a function of strategy. Using the strategy analyses introduced by Gluck et al. (2002) and described above, they dichotomized participants into those using one-cue or singleton strategies (who they refer to as *declarative learners*), and those using a multi-cue strategy (who they refer to as *nondeclarative learners*). They compared electrophysiological responses (FRN and P300) to feedback between strategy groups. The authors hypothesized that those who used the multi-cue strategy were engaging implicit systems and would experience larger FRN amplitudes than those who used one-cue or singleton strategies, as the FRN is thought to reflect dopaminergic reward input to the anterior cingulate cortex, which is a key component of implicit reward-mediated learning (Holroyd and Coles, 2002; Eppinger et al., 2009; Hauser et al., 2014). They also hypothesized that those who used a one-cue or singleton strategy were engaging in declarative systems and would experience larger P300 amplitudes than those who used a multi-cue strategy, as the P300 is thought to reflect a more declarative learning approach/process (Rustemeier et al., 2013). They did not find strategy associations with the FRN, which they conclude may have been due to an insufficient number of strategy classifications, and state that the addition of a third mixed strategy may have yielded clearer group differences in the FRN. Nonetheless, this work set a foundation for utilizing the FRN to better understand how feedback processing might relate to strategy development and use. The authors did, however, find a strategy association with the P300. Before detailing their P300 findings, it is important to note that Rustemeier et al. (2013) describe a more pronounced P300 originating from the frontal electrode, Fz, which is likely and more specifically, the P3a (Friedman et al., 2001; Butterfield and Mangels, 2003). The authors found that those who used one-cue or singleton strategies exhibited a larger P300 (P3a) than those who used the multi-cue strategy, and concluded that this reflected differential neural mechanisms involved in feedback processing between their declarative and nondeclarative learners (Rustemeier et al., 2013).

In sum, researchers have measured learning accuracy and conducted strategy analyses to gather information specific to learning outcomes. These works have increased the field’s understanding of humans’ approaches to and success with learning. To further elucidate what occurs *during* the learning process and how the provision of feedback affects the learning process, researchers have utilized the ERP methodology. Such work has increased the field’s understanding of feedback-based learning, as well as how individual differences in feedback processing may relate to and help predict learning outcomes. Less studied, however, is how individual differences in feedback processing may relate to learning approach, i.e., learning strategy, and how the provision of feedback influences strategy development. While work in this area continues to emerge, there is a notable lack of studies that have combined behavioral and electroencephalographic measures to study the contribution of feedback to the development of learning strategies. The novelty of the present study is in its evaluation of changes in feedback processing at the electrophysiological level as they relate to the development of learning strategies.

The objective of the current study was to examine the relationships between learning condition, learning success, and strategy development during a complex, probabilistic category learning task performed by the same participants under two conditions. Additionally, the study set out to explore the potential interactions between strategy development and measures of feedback processing. To do so, the study examined performance on a probabilistic A/B prototype category learning task in young adults with no history of neurologic disorder. Participants completed observational training (i.e., no feedback) followed by a testing phase, and feedback-based training, also followed by a testing phase. In analyzing the results, the researchers compared testing accuracy following observational and feedback-based training. Further, the researchers examined the strategies employed by the learners during both testing phases, as well as the strategies they employed during training in the feedback-based condition. Within the feedback-based training condition, the researchers also examined how learners processed positive and negative feedback during the early and late training phases *via* electrophysiological measurement of the FRN and P3a.

Behaviorally, the researchers expected participants to achieve higher testing accuracy following feedback-based training, as prior research suggests feedback-based training (where the correct category classification is shown after the stimulus) is more effective for learning complex categories that require the integration of multiple stimulus dimensions than observational training (Estes, 1994; Ashby et al., 2002). The researchers also expected to see a variety of strategy use, with a greater number of multi-cue strategy users following feedback-based training, because the provision of feedback is thought to be important for the development of multi-dimensional strategies (Poldrack et al., 2001; Ashby et al., 2002). Consistent with the literature, the researchers expected negative feedback to elicit larger FRN and P3a amplitudes than positive feedback (Holroyd and Coles, 2002; Butterfield and Mangels, 2003; Ernst and Steinhauser, 2012; Arbel et al., 2013; Rustemeier et al., 2013). Beyond these expected findings, the researchers predicted that multi-cue strategy users would demonstrate larger FRN amplitudes, which would reflect feedback utilization for optimization and reward prediction error processing. Based on the findings by Rustemeier et al. (2013), the researchers predicted that single feature and random pattern strategy users would elicit larger P3a amplitudes than multi-cue users, which would reflect the formation of explicit associations between stimuli and responses, and would be consistent with a more rule-based and declarative approach.

## Materials and Methods

### Participants

Thirty-eight young adults (self-identified: 25 women, 10 men, and three unknown) with a mean age of 25years (SD=3.33) from the greater Boston area participated in this study. Racial and ethnic demographic information was not collected from the participants at the time of data collection. Participants provided informed consent according to processes approved by the Mass General Brigham Institutional Review Board. All participants self-reported to be right-handed with no history of developmental disorders or neurological diagnoses.

### Apparatus

To collect and analyze EEG data, the researchers used the Geodesic EEG System (GES) 400 by Electrical Geodesics, Inc. (EGI) with a 32-channel HydroCel Geodseic Sensor Net that followed the international 10–20 system. EEG was recorded continuously at a 1,000Hz sampling rate. Electrode impedances were kept below 50kΩ.

### Stimuli

**Figure 1:**
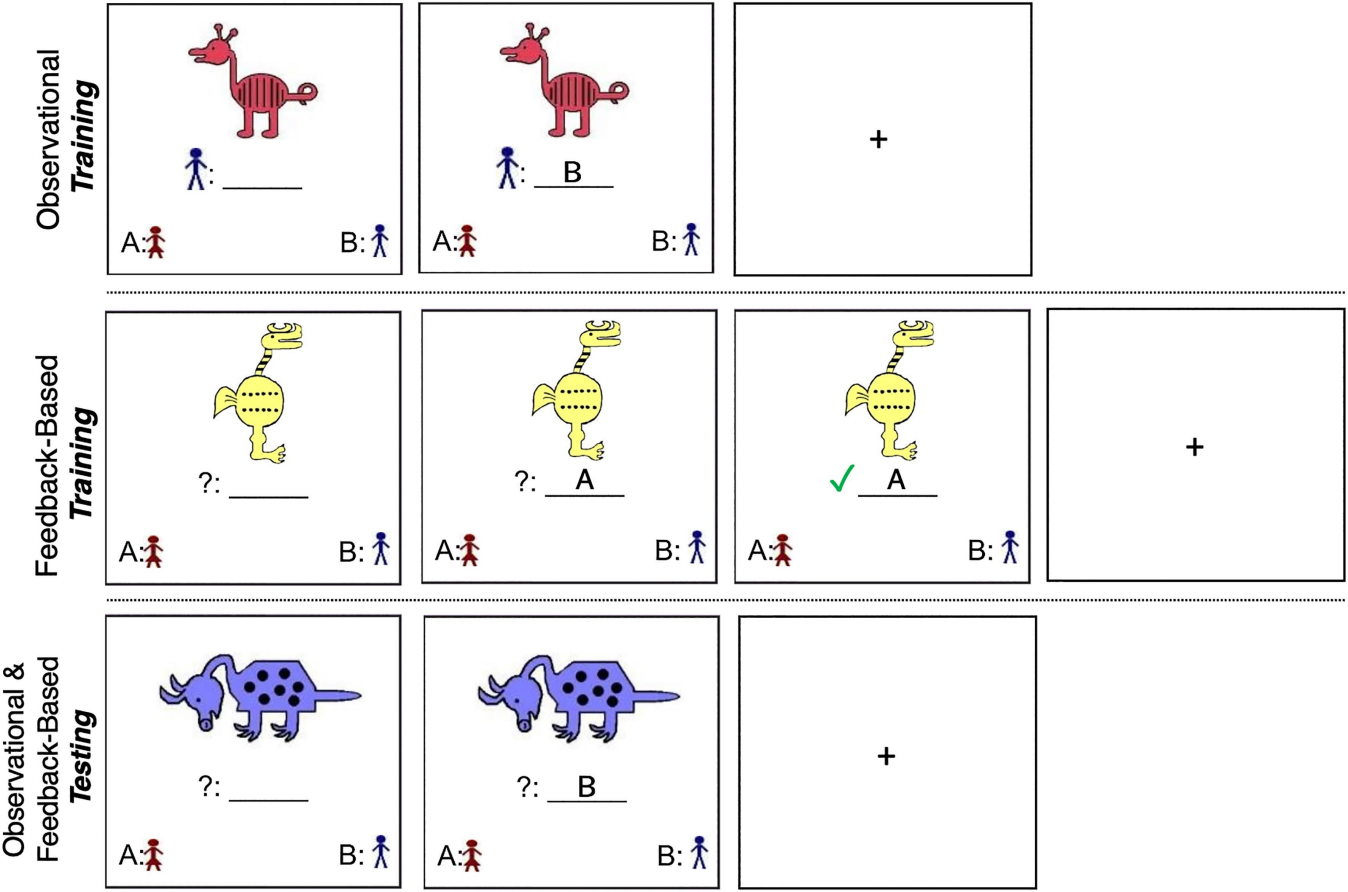
(Top) Example of the observational training phase (Stimulus from Set 1; Reed et al., 1999; Zeithamova et al., 2008). Participants saw a stimulus and its category affiliation simultaneously, followed by a fixation cross between each trial. (Middle) Example of the feedback-based training phase (Stimulus from Set 2). Participants saw a stimulus, selected to which category they thought it belonged, and received feedback on their selection. (Bottom) Example of the testing phase (Stimulus from Set 1). Participants saw a stimulus, selected to which category they thought it belonged, and did not receive feedback.

The stimuli used for this experiment consisted of two sets of cartoon animals, which were first introduced by Reed et al. (1999) and updated by Zeithamova et al. (2008). The 1,024 cartoon animals varied on 10 binary features, including features such as leg length, nose shape, foot type, and so on. For example, the animals could either have pointed feet or rounded feet (for examples of stimuli, see Figure 1). There were two different stimulus sets used for this experiment, referred to as Stimulus Set 1 and Stimulus Set 2, that were completely distinct and whose stimuli did not share any features. Two prototypes, completely opposite of each other in all 10 features, were chosen for each Stimulus Set and were referred to as Prototype A and Prototype B. All other animals were then characterized by the number of features by which they differed from Prototype A. An animal characterized as being at a distance of four from Prototype A, for example, shared all but four of its features with Prototype A (meaning that it shared six of its features with Prototype A). The four features that it did not share with Prototype A, it shared with Prototype B. Prototype B is the only animal that differed from Prototype A by all 10 of its features, giving it a distance of 10.

Animals at a distance of one, two, three, or four from Prototype A, shared 90–60% of their features with Category A, respectively, and were coded as belonging to Category A. Similarly, animals at a distance of six, seven, eight, or nine were coded as belonging to Category B. Animals at a distance of five shared equal features with the two prototypes and were therefore not shown in training. A 10-digit string based on the particular animal’s features was assigned to each animal in the set, with binary features represented as 0 or 1 (0 denoting a Category A feature, and 1 denoting a Category B feature).

### Tasks and Procedures

The study used a quasi-experimental design. Participants visited the lab once for a 1-h long session. Trained members of the research team applied the 32-channel HydroCel Net on the participant’s scalp and recorded EEG while participants completed the learning task under two different conditions. Participants completed the task sitting in front of a 15-inch computer screen in a quiet room at the MGH Institute of Health Professions after instruction by a trained task administrator and made responses *via* keyboard clicks. After the nets were applied and impedances were adjusted, the task administrator provided verbal instructions regarding the task, accompanied by illustrated pictures. Participants were told that they would be shown a series of different animals, which either belonged to Category A or Category B, and that they would learn to recognize the animals as belonging to one of the two categories throughout the course of the task. Participants were told not to focus on just one or two of the animal’s features, but the entire animal. To support comprehension of the task, participants were allowed to ask questions and administrators could repeat instructions or provide additional examples verbally and with pictures. Once participants communicated that they understood the task instructions, they moved on to computerized training that reinforced task instructions. The two task conditions were programmed using E-Prime 2.0 (Psychology Software Tools, Pittsburgh, PA; Psychology Software Tools, Inc., 2012),^1^ which was also used to present the stimuli and record responses. Four tasks were created: observational learning of Stimulus Set 1, observational learning of Stimulus Set 2, feedback-based learning of Stimulus Set 1, and feedback-based learning of Stimulus Set 2. Each task consisted of an 80 trial, 10-min training phase followed immediately by a 50 trial, 10-min testing phase. All participants were instructed to make responses *via* computer button press with the middle and index fingers of their right hand. All participants completed the observational version of the task (with Stimulus Set 1 or 2) first followed by the feedback-based version (with the opposite Stimulus Set they saw in the observational task), both of which will be described below. To keep the observational condition free of any outcome prediction that might result from exposure to the feedback-based (outcome generating) task, participants always completed the observational condition first. Participants completed one round of each condition and the researchers counterbalanced Stimulus Sets across participants.

#### Observational Training

In the training phase of the observational task, Category A and Category B animals were presented one at a time along with a label indicating the animal’s designated category affiliation. Participants were instructed to press the button that corresponded to the label immediately after the animal and its accompanying category label appeared on the screen (see Figure 1). They were told that the animal and its category label would remain on the screen for a fixed number of seconds (7,000ms, followed by a 1,000ms fixation cross), and were instructed to examine the animals and their category affiliation with the goal of later recognizing the animals as belonging to one category or the other. In examining the animals, participants were instructed to consider all of their characteristics rather than focus on one single feature. Vallila-Rohter and Kiran (2013) include additional details regarding these methods.

During the 80 trial, 10-min training phase, participants were shown 20 different animals, four times each. Ten of the 20 animals differed from Prototype A by one, two, three, or four features, and the other 10 animals differed from Prototype A by six, seven, eight, or nine features (meaning that they differed from Prototype B by one, two, three, or four features). The two prototypical animals were never shown. Features that were characteristic of a category were associated with that category in 70–80% of instances, and features that were not characteristic of a category were associated with that category in 20–30% of instances. Immediately following the training phase, participants completed a testing phase (described below in *Testing Phases*) that tested their ability to categorize animals, some of which were seen during training and some of which were novel.

#### Feedback-Based Training

Similar to the observational task, Category A and Category B animals were randomly presented one at a time on a computer screen. Once an animal appeared on the screen, participants were given 4,000ms to guess to which of the two categories that animal belonged. Pictures and category identifiers in the lower left and right corners of the screen indicated that a button press “A” corresponded to a choice of Category A and a button press “B” corresponded to a choice of Category B. Participants received feedback telling them whether their selection was correct or incorrect 500ms after making their response (see Figure 1). Feedback was displayed for 3,000ms. If they made an incorrect choice, the correct category was shown during the 3,000ms post-response feedback period. Total time per trial matched the total trial time of the observational task.

During the 80 trial, 10-min training phase, participants were trained on 20 animals that differed from each prototype by one, two, three, or four features. The two prototypical animals were never shown. Trained animals were selected so that each feature appeared an equal number of times during training. Features that were characteristic of a category were associated with that category in 70–80% of instances, and features that were not characteristic of a category were associated with that category 20–30% of instances. Participants were instructed to consider all of an animal’s characteristics rather than focusing on single features. They were told that in the beginning of the task, they would be guessing category affiliations, but that over the course of the task they would begin to recognize animals as belonging to one category *via* feedback and practice. Immediately following the training phase, participants completed a testing phase that tested their ability to categorize animals, some of which were seen during training and some of which were novel. No feedback was given during the testing phase.

#### Testing Phases

The testing phases immediately following the observational and feedback-based training phases were structured identically, each with 50 trials. The animal stimuli appeared one at a time on the computer screen. Ten of the animals were seen during training and 40 were novel members of the categories, which included the two prototypical animals. Participants were tested on their ability to categorize each prototype two to three times each for a total of five to six trials, animals that varied from Prototypes A and B by one to four features for a total of 40 trials (including 10 seen during training), and midline animals varying from each prototype by five features for a total of two trials. These midline animals have no correct category as they share an equal number of features with each prototype, and were coded as belonging to Category A, with expected response accuracy for these midline animals to be approximately 50%.

Participants were given 4,000ms to select to which category they thought the animal belonged. If a participant waited too long to make a selection or missed a trial due to a delay in selection that exceeded the allotted 4,000ms, they were encouraged by the testing administrator to make a button press indicative of their best guess. No feedback was provided during the testing phases. Data were collected on accuracy and response time. For the current paper, analyses are limited to accuracy rates, as analyses of response time were outside of the scope of this paper. In terms of testing accuracy, the researchers predicted that the participants would make responses that roughly reflected characteristic reinforcement as seen during training due to the probabilistic nature of the task (Knowlton et al., 1994). Chance responses would result in 50% accuracy, and responses that mirrored characteristic reinforcement would result in ~70–80% mean testing accuracy.

### Strategy Analysis

For additional characterization of learning, the researchers conducted strategy analyses on each participant’s data to examine response patterns during feedback-based training, observational testing, and feedback-based testing. Observational training was excluded from this analysis because during observational training, participants simply pressed the corresponding button to the correct response shown on the screen, which did not require them to make trial-by-trial decisions as they did in the other three task phases. For the strategy analyses, the researchers examined trial-by-trial responses based on response selection (A or B) as they related to individual features. Using the models presented by Gluck et al. (2002) and Meeter et al. (2006), the researchers created multiple model strategies adapted to the two tasks and stimuli used for this study. There were 22 models in total; one optimal, multi-cue strategy, 20 single feature strategies, and one random pattern strategy. Recall that every animal had 10 features that could vary binarily, with one possibility coded as Category A and the other coded as Category B. The researchers examined the percentage of “B” responses made for each binary feature (e.g., the percentage of times a participant select “B” when they saw an animal with a blue body, and the percentage of times they selected “B” when they saw an animal with a yellow body).

The optimal, multi-cue strategy reflected a response pattern that matched the actual “B” reinforcement rate seen during training. For example, participants were shown animals with a blue body as belonging to Category A in 20% of instances to Category B in 80% of instances during training. For all 10 binary features, the multi-cue strategy modeled response patterns that matched each feature’s reinforcement rate during training. Actual response rates that matched optimal categorization for multiple features resulted in a best fit to the multi-cue strategy. The single feature strategies reflected response patterns that revealed reliance on the presence or absence of a single feature. Two single feature strategy models were built for each of the 10 features. For example, the A-single feature strategy for the feature “blue body” modeled responses in which the participant responded with “A” in 95–100% of trials in which a blue body was shown on the screen. The B-single feature strategy for blue body modeled responses in which the participant responded with “B” in 95–100% of trials in which a blue body was shown on the screen. Finally, the random pattern strategy reflected a response pattern that modeled a 50% B-response rate to each feature dimension and is thought to represent either no feature-focused strategy, random behavior, or a variety of strategies that deviate from multi-cue and single-feature (Meeter et al., 2006; Vallila-Rohter and Kiran, 2015). The inclusion of a random model in strategy analyses helps to reduce the number of falsely identified multi-cue and single feature strategy fits (Meeter et al., 2006). The study used the quantitative methods proposed by Gluck et al. (2002) and adapted by Vallila-Rohter and Kiran (2015) to quantify the fit of each participant’s responses to each possible model. The researchers used the following calculation to assign each participant with a fit score for each model:


$$\text{Score for Model }M=\frac{{\text{Σ}}_{F}\;{\left(\#B\;{\text{expected}}_{F,M}-\#B\;{\text{actual}}_{F}\right)}^{2}}{{\text{Σ}}_{F}{\left(\#B\;{\text{presentations}}_{F}\right)}^{2}}$$

*F* indicates the feature (10 features, all with binary values); #*B* expected*_F,M_* indicates the amount of times a B-response would be expected for each feature under Model *M* based on reinforcement during training; #*B* actual*_F_* indicates the number of B-responses for each feature; and #*B* presentations*_F_* indicates the amount of times the feature B appeared in testing. The researchers calculated a fit score between 0 and 1 for each strategy model for each participant. The fit score closest to 0 represented the closest match with ideal model data. Each participant’s response data were separated into: early feedback-based training (training trials 1–40), late feedback-based training (training trials 41–80), observational testing, and feedback-based testing, and the researchers conducted strategy analyses for all four.

Implementing a multi-cue strategy requires attention to multiple features at once and requires the tracking of feedback and the acquisition of cue-outcome relationships across multiple dimensions at one time (Vallila-Rohter and Kiran, 2015). Considering the probabilistic nature of the task, the multi-cue strategy was expected to lead to higher testing accuracy scores (Ashby et al., 2002). Implementing a single feature strategy would mean that the participant determined their responses based on the presence or absence of single features. While these strategies can lead to adequate learning (i.e., above-chance performance) if participants attend to a feature dimension with a high proportion of reinforcement with a category, single feature strategies are often described as suboptimal because selections are based on the presence of a single entity (Shohamy et al., 2004), and focusing on single features does not require the tracking of feedback across multiple features at once (Vallila-Rohter and Kiran, 2015). As previously stated, the implementation of a random pattern strategy is thought to represent either no feature-focused strategy, random behavior, or a variety of strategies that deviate from multi-cue and single-feature (Meeter et al., 2006; Vallila-Rohter and Kiran, 2015).

### ERP Data

The 32-channel GES 400 System by EGI was used to collect and analyze EEG data for 37 of the 38 participants. One participant’s EEG data were lost due to system error. EEG data were filtered offline using a bandpass of 0.1–30Hz. The filtered data were segmented into epochs 1,000ms in duration; 200ms before and 800ms after the presentation of feedback during the feedback-based training phase only. Baseline correction from −200 to −100ms was performed. All epochs were visually inspected for movement artifacts and any movement artifacts were removed manually. The researchers used an algorithm developed by Gratton et al. (1983) to remove ocular artifacts offline to adjust for blinks and other eye movements. Averages were then re-referenced using average referencing. Following a jitter latency correction, temporal principal component analysis was performed (PCA; Dien et al., 2005; Arbel and Wu, 2016; Arbel et al., 2017, 2021) on the ERPs from the fronto-central recording electrode (FCz) to isolate the FRN and the P3a. Seven temporal factors accounted for 88% of the variance in the data. Temporal factor 6 peaked at approximately 248ms, reflective of FRN activity. Temporal factor 4 peaked at approximately 360ms, reflective of P3a activity. The PCA yielded FRN and P3a factor scores for positive and negative feedback (termed: *feedback valence*) during both early and late training trials (termed: *training phase*).

**Figure 2:**
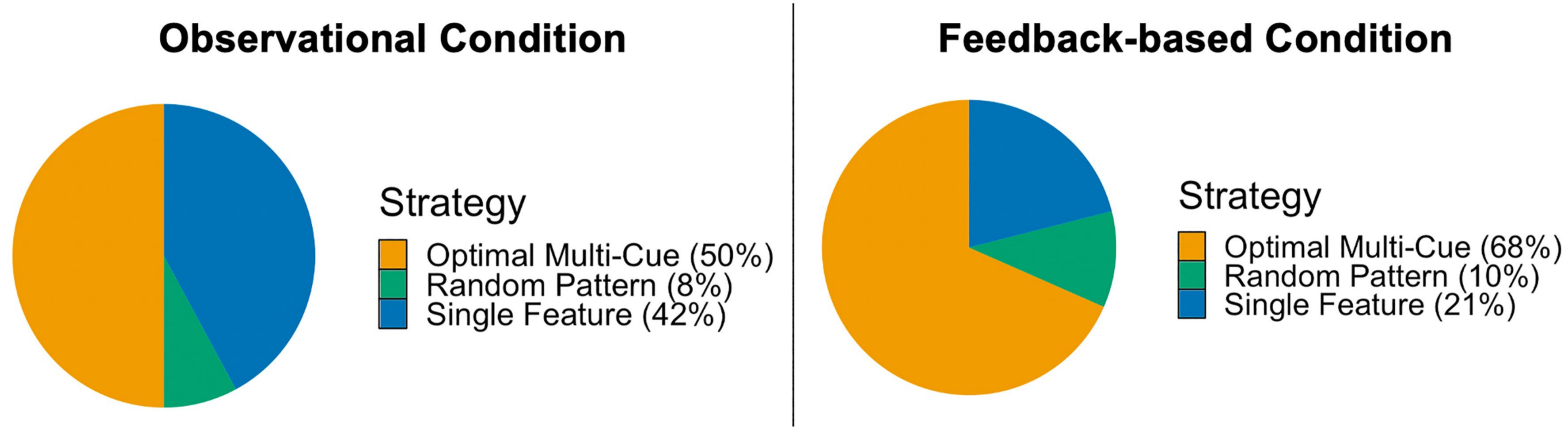
A chi-square goodness of fit indicated that the strategy proportions under the two task conditions were significantly different, *χ*^2^(2)=6.85, *p*=0.03. Feedback-based training resulted in a larger proportion of multi-cue users and a smaller proportion of single feature users in the testing phase than observational training.

**Figure 3:**
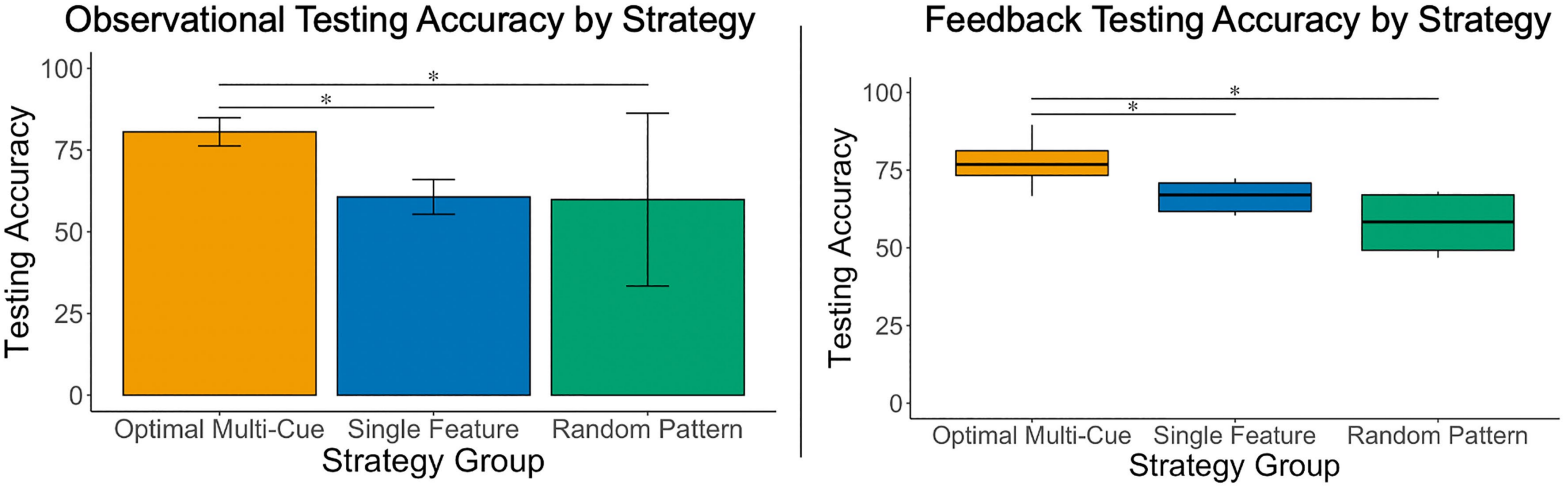
A one-way, between-subjects ANOVA and a Kruskal–Wallis test revealed that participants who used an optimal multi-cue strategy achieved significantly greater testing accuracy than those who used single feature and random pattern strategies, in both the observational and feedback-based conditions. Results did not indicate significant differences in testing accuracy between participants who used a single-feature or random pattern strategy in either condition. *p<0.05.

The researchers conducted the PCA using MATLAB and all statistical analyses using R (R Core Team, 2020) in RStudio (RStudio Team, 2019). The colorblind-friendly color palette used to create Figures 2, 3 was found at: www.cookbook-r.com/Graphs/Colors_(ggplot2)/#a-colorblind-friendly-palette.

## Results

### Preliminary Analysis

An independent samples t-test confirmed that there were no significant differences in mean testing accuracy between Stimulus Set 1 and Stimulus Set 2 under the observational condition, *t*(36)=−0.59, *p*=0.56, 95% CI [−11.78, 6.46] and the feedback condition, *t*(36)=0.14, *p*=0.89, 95% CI [−5.73, 6.56]. Therefore, data across Stimulus Set 1 and 2 were collapsed for subsequent analyses.

### Testing Accuracy

The researchers conducted a dependent samples *t*-test to examine whether there was a significant difference in mean testing accuracy scores achieved under the observational and feedback-based conditions. The assumptions of normality of difference scores and independence were met. The difference in mean testing accuracy scores achieved under the observational (*M*=69.65, SD=13.67) and feedback-based (*M*=73.24, SD=9.17) conditions was not significant, *t*(37)=−1.56, *p*=0.13, 95% CI [−8.25, 1.08], *d_av_*=−0.31, 95% CI [−0.64, 0.01].

### Strategy Analysis

During the observational testing phase, 50% (*n*=19) of the participants used a multi-cue strategy, 42% (*n*=16) used a single feature strategy, and 8% (*n*=3) used a random pattern strategy. Under the feedback-based testing phase, 68% (*n*=26) used a multi-cue strategy, 21% (*n*=8) used a single feature strategy, and 10% (*n*=4) used a random pattern strategy (see Figure 2). A chi-square goodness of fit test indicated that the strategy proportions under the two task conditions were significantly different, *χ*^2^(2)=6.85, *p*=0.03. Feedback-based training resulted in a larger proportion of multi-cue users and a smaller proportion of single feature users in the testing phase than observational training.

### Testing Accuracy by Strategy

#### Observational Condition

The researchers conducted a one-way, between-subjects analysis of variance (ANOVA) to examine whether there were significant differences in mean testing accuracy scores depending on the participants’ testing strategy (Strategy: multi-cue vs. single feature vs. random pattern) under the observational condition. Levene’s test was not significant, *p*=0.58. There were significant differences in mean testing accuracy scores depending on the participants’ testing strategy, *F*(2, 35)=25.09, *p*<0.001, *η*^2^=0.59. *Post-hoc* analyses using a Holm adjustment revealed that mean testing accuracy scores were higher for participants using a multi-cue strategy (*M*=80.0, SD=7.27) relative to those using a single feature strategy (*M*=59.2, SD=10.52), *p*<0.001, *d_s_*=2.34, 95% CI [1.46, 3.20], and those using a random pattern strategy (*M*=59.86, SD=10.65), *p*<0.01, *d_s_*=2.62, 95% CI [1.14, 4.06] (see Figure 3), all of which is consistent with expectations. The comparison between those using a single feature vs. random pattern strategy was not statistically significant, *p*=0.91, and was associated with a marginal effect, *d_s_*=−0.06, 95% CI [−1.29, 1.17].

#### Feedback-Based Condition

The researchers conducted a Kruskal–Wallis test rather than a parametric ANOVA, due to heterogeneity of variances in testing accuracy between strategy groups, to examine differences in mean testing accuracy scores depending on the participants’ testing strategy (Strategy: multi-cue vs. single feature vs. random pattern) under the feedback-based condition. There were significant differences in mean testing accuracy scores depending on the strategy, *H*(2)=21.62, *p*<0.001, with a mean rank accuracy score of 25.15 for multi-cue users, 8.38 for single feature and 5.0 for random pattern users (with higher mean ranks indicating higher accuracy score ranks). Similar to the observational condition results, *post-hoc* analyses using a Holm adjustment demonstrated that participants using a multi-cue strategy (Mdn=76.84, IQR=7.95) achieved higher scores than those using a single feature strategy (Mdn=66.48, IQR=9.13), *p*<0.01 and those using a random pattern strategy (Mdn=58.33, IQR=17.82), *p*<0.01 (see Figure 3), all of which is consistent with expectations. The comparison between those using a single feature vs. random pattern strategy was not statistically significant, *p*=0.23.

### Event Related Potentials Analyses

**Figure 4:**
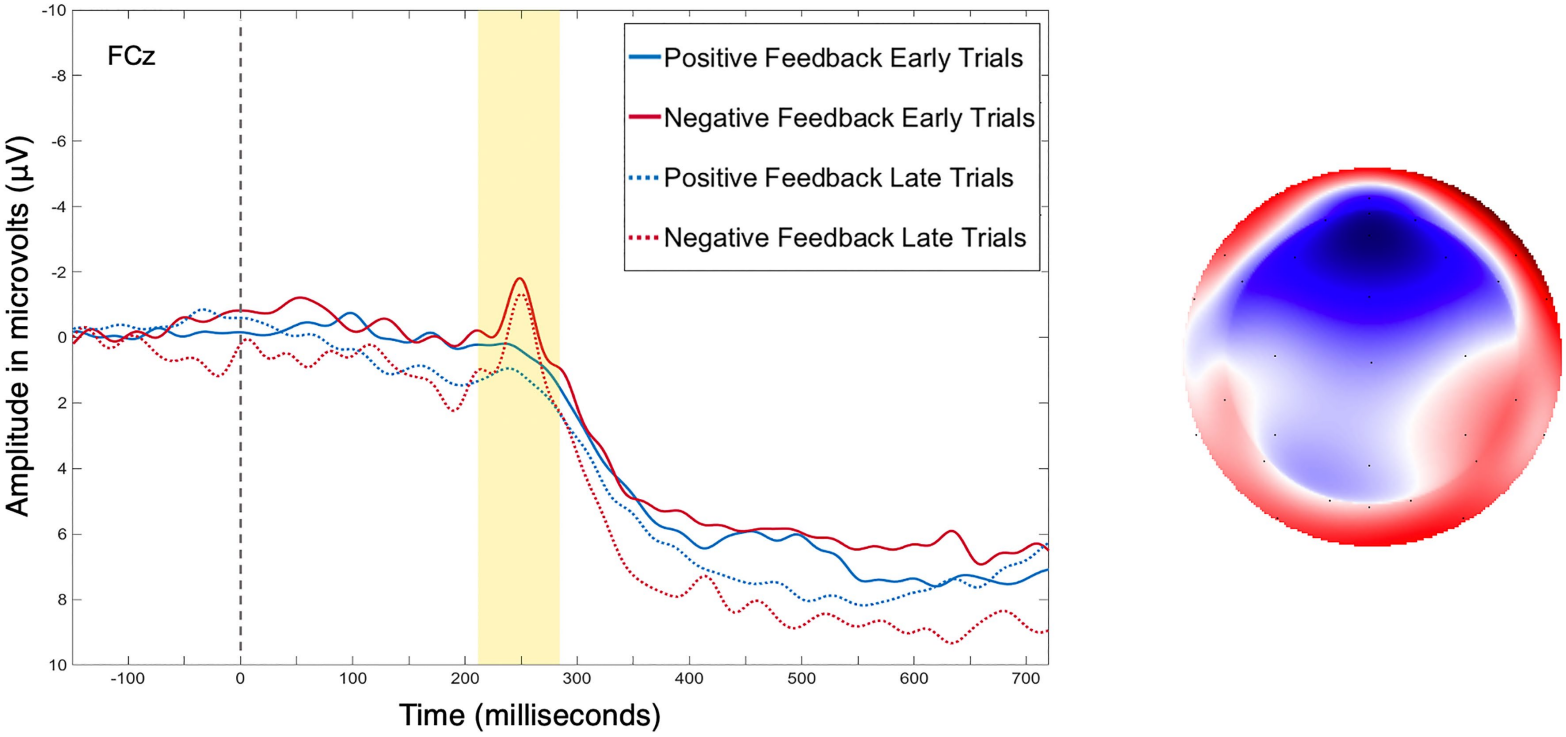
(Left) As is standard with ERP plotting, the y-axis has negative amplitude values plotted upwards. Grand average event-related potentials (ERP) data from electrode FCz, for positive and negative feedback during early (trials 1–40) and late (trials 41–80) training trails. (Right) Topoplot depicting the difference between positive and negative feedback at the peak of the feedback-related negativity (FRN).

Figure 4 presents the grand average waveform from electrode FCz where the FRN and P3a are maximal. The factor scores of temporal factor 6 generated by the temporal PCA were entered into the analysis as the amplitude measure of the FRN, and factor scores of temporal factor 4 generated by the temporal PCA were entered into the analysis as the amplitude measure of the P3a.

#### Feedback Frequency

To ensure that all participants received an adequate number of negative feedback trials in early and late training to detect a significant ERP effect, the researchers calculated descriptive statistics on the mean, standard deviation, minimum, and maximum values of negative feedback trials in early and late training (*n*=37). In early training, participants received an average of 13.58±3.64, a minimum of 7, and a maximum of 22 incorrect feedbacks. In late training, participants received an average of 10.84±5.06, a minimum of 2 and a maximum of 23 incorrect feedbacks. Studies show that a minimum of six negative feedback trials can be adequate with a minimum *n* of 12 in order to detect a significant ERP effect (Boudewyn et al., 2018). Six participants received less than six instances of negative feedback, therefore, their data were excluded from the ERP analyses. With those six participants excluded (*n*=31), the researchers recalculated descriptive statistics. In early training, participants received an average of 14.49±3.56, a minimum of 7, and a maximum of 22 instances of negative feedback. In late training, participants received an average of 12.42±4.14, a minimum of 6, and a maximum of 23 instances of negative feedback.

#### Changes in Feedback Frequency

Individuals’ processing of feedback is largely impacted by the frequency of the feedback itself (in this case, the frequency of negative and positive feedback; Holroyd et al., 2003; Rustemeier et al., 2013). Therefore, the groups’ changes in frequency of negative (and therefore positive) feedback from early to late training were compared, to further contextualize the ERP results and increase confidence that ERPs were not significantly modulated by reward frequency (Holroyd et al., 2003; Rustemeier et al., 2013). First, each participants’ number of negative feedback trials in late training was subtracted from their number of negative feedback trials in early training. This value became their “change in negative feedback score,” in which a positive value indicated that they received less negative feedback in late training than they did in early training (which would be expected with learning), and a negative value indicated that they received more negative feedback in late training compared to early training. Then, the researchers conducted a factorial ANOVA comparing the mean changes in negative feedback between the three strategy groups. Levene’s test was not significant, *p*=0.16. The ANOVA revealed no significant group differences in the change in negative feedback trials from early to late training, *F*(2, 28)=0.63, *p*=0.54.

#### Feedback-Related Negativity

The researchers conducted a 2 (Feedback valence: positive vs. negative) by 2 (Training phase: early vs. late) by 3 (Strategy: multi-cue vs. single feature vs. random pattern) mixed-design ANOVA to examine whether the effects of feedback valence and training phase on FRN amplitude varied depending on strategy. While group sizes differed for multi-cue (*n*=19), single feature (*n*=8), and random pattern strategy users (*n*=4), Levene’s test, assessing homogeneity of variance for the between-subjects independent variable (Strategy) was not significant, *p*=0.31. As recommended by Larson and Carbine (2017), correlations between repeated measures were calculated. The correlation between FRN amplitude following positive feedback in early training and FRN amplitude following positive feedback in late training equaled 0.38. The correlation between FRN amplitude following negative feedback in early training and FRN amplitude following negative feedback in late training equaled 0.29.

**Figure 5:**
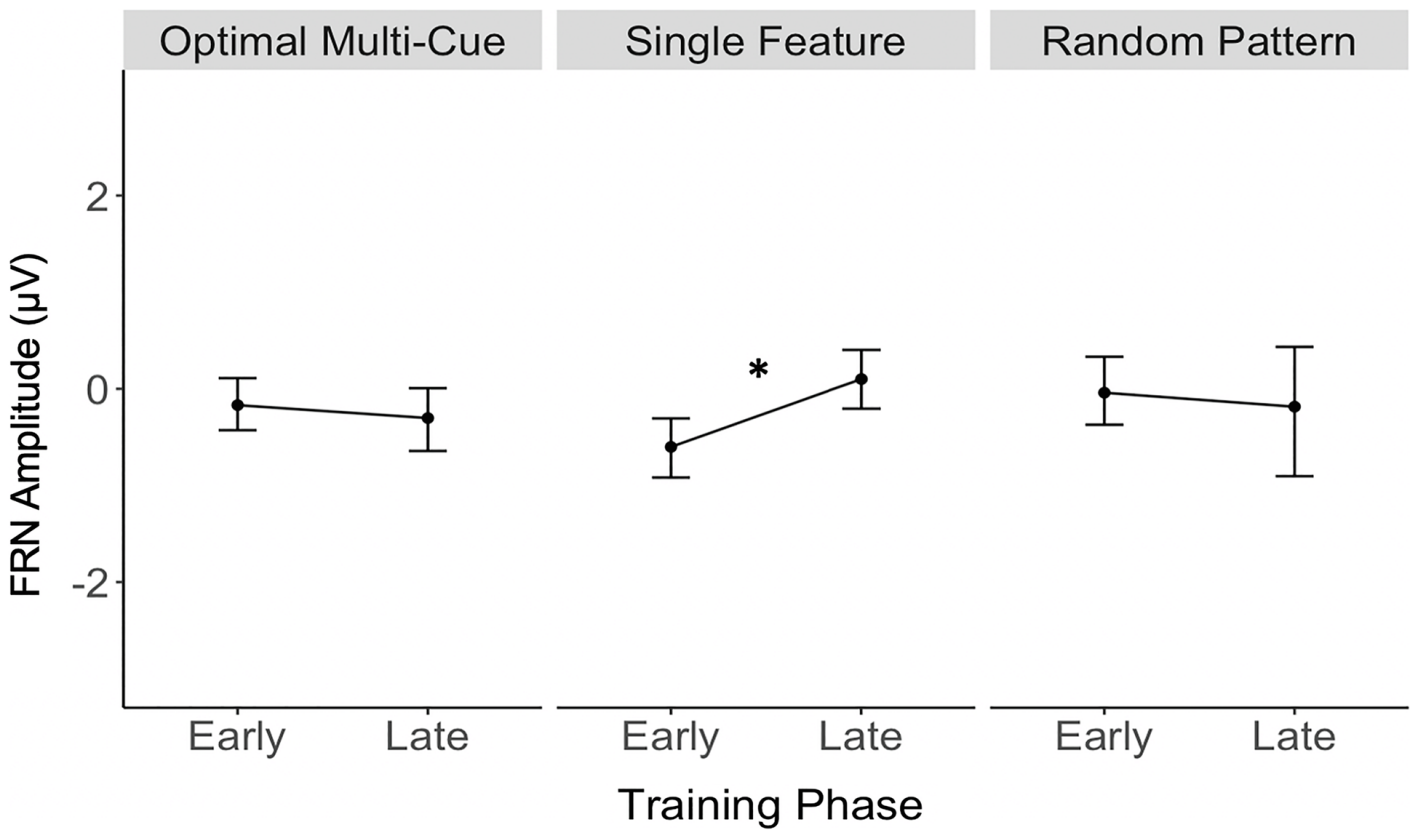
Single feature strategy users’ FRN amplitude was significantly smaller (i.e., became less negative) in late training compared to early training. *p<0.05.

Table 1 contains a complete summary of the mixed-design ANOVA results. There was a significant main effect of feedback valence on FRN amplitude, *F*(1, 28)=7.55, *p*=0.01, *η*^2^=0.05, with FRN amplitude to negative feedback (*M*=−0.38μV, SD=0.88) significantly larger than to positive feedback (*M*=−0.07μV, SD=0.88), *d_av_*=−0.36, 95% CI [−0.62, −0.10]. There was also a significant two-way interaction between training phase and strategy, *F*(2, 28)=3.63, *p*=0.04, *η*^2^=0.05. Results of *post-hoc* contrasts calculated using the “emmeans” package (Lenth, 2019) revealed that the single feature strategy group experienced a significant change in FRN amplitude from early to late training, *t*(28)=−2.62, *p*=0.01. More specifically, their FRN amplitude was significantly smaller (i.e., it became less negative) in late training (*M*=0.08μV, SE=0.24) than in early training (*M*=−0.63μV, SE=0.24), which was associated with a large effect (Cohen, 1988), *d_av_*=−2.86, 95% CI [−3.97, −1.72], as shown in Figure 5. Alternatively, the multi-cue strategy group did not experience a significant change in FRN amplitude from early (*M*=−0.19μV, SE=0.2) to late (*M*=−0.33μV, SE=0.2) training, *t*(28)=0.77, *p*=0.44, *d_av_*=0.66, 95% CI [0.30, 1.01], nor did the random pattern strategy group (*M*_early_=−0.06μV, SE=0.3), (*M*_late_=−0.21μV, SE=0.3), *t*(28)=0.38, *p*=0.70, *d_av_*=−0.48, 95% CI [−0.27, 1.20].

**Table 1:** Feedback-related negativity amplitude, 2×2×3 ANOVA table.

Predictor	*df*_Num_	*df*_Den_	SS_Num_	SS_Den_	*F*	*p*	*η* ^2^_g_
(Intercept)	1	28	3.35	39.07	2.40	0.132	0.04
Strategy	2	28	0.23	39.07	0.08	0.921	0.00
Training phase	1	28	0.42	16.02	0.73	0.401	0.01
Feedback valence	**1**	**28**	**4.03**	**14.94**	**7.55**	**0.010**	**0.05**
Strategy×Training phase	**2**	**28**	**4.15**	**16.02**	**3.63**	**0.040**	**0.05**
Strategy×Feedback valence	2	28	2.86	14.94	2.68	0.086	0.04
Training phase×Feedback valence	1	28	0.53	7.39	1.99	0.169	0.01
Strategy×Training phase×Feedback valence	2	28	1.44	7.39	2.72	0.083	0.02

*There was a significant main effect of feedback valence, F(1, 28)=7.55, p=0.01, η^2^=0.05, and bold values indicate the significant main effect of feedback valence and the significant two-way interaction between training phase and strategy*.

#### P3a

The researchers conducted a 2 (Feedback valence: positive vs. negative) by 2 (Training phase: early vs. late) by 3 (Strategy: multi-cue vs. single feature vs. random pattern) mixed-design ANOVA to examine whether the effects of feedback valence and training phase on P3a amplitude varied depending on strategy. The correlation between P3a amplitude following positive feedback in early training and P3a amplitude following positive feedback in late training equaled 0.78. The correlation between P3a amplitude following negative feedback in early training and P3a amplitude following negative feedback in late training equaled 0.52. Levene’s test was not significant, *p*=0.48. The ANOVA results revealed no significant main effects or interaction effects of feedback valence, training phase, or strategy on P3a amplitude.

## Discussion

Results of the current study add to the body of work that suggests that humans approach the process of categorization in multiple ways (see Ashby and Maddox, 2011). Consistent with prior work (Gluck et al., 2002; Meeter et al., 2006; Shohamy et al., 2008; Vallila-Rohter and Kiran, 2015), strategy analyses revealed that participants approached this multi-dimensional category learning task with a variety of strategies. Even though the probabilistic, multi-dimensional nature of the stimuli used in this study necessitated information accrual across multiple dimensions for optimal learning and made it such that information from a single trial was not reliable or sufficient to give the learner a full representation of the category boundary, some participants still tried to apply verbalizable, single-feature rules when making responses. The WPT, a probabilistic task utilized in many research studies, has often been assumed to be learned in a certain manner. Current findings are consistent with studies acknowledging that participants vary in their approach to learning probabilistic tasks (Gluck et al., 2002; Meeter et al., 2006; Vallila-Rohter and Kiran, 2015). Thus, even if a structure or task is optimally learned a certain way, learners may vary in their approach to learning.

Also consistent with prior studies (Ashby et al., 2002), feedback-based training resulted in a greater number of optimal, multi-cue strategy users than observational training, which provides additional evidence of the importance of feedback on the development of multi-dimensional strategies. While overall accuracy was relatively matched between the two conditions, strategies differed. Previous work has shown that providing trial-by-trial feedback results in an ability to learn complex category rules that are non-verbalizable, and without it, people show a tendency to use simple and verbalizable rules (Ashby et al., 1998). It may be that the dopaminergic reward-mediated system which is well-suited for integrating across multiple dimensions is best recruited when learners engage in a process of rewarded or un-rewarded prediction. Such prediction is less likely to arise in observational contexts where correct responses are provided to a learner than those in which a learner is required to generate a response before receiving feedback or the correct response. Therefore, findings provide additional support that although different learners will employ different strategies even when completing the same task, the provision of trial-by-trial feedback may result in a higher likelihood of learners developing an optimal, multi-dimensional strategy. In terms of accuracy, those identified as having used an optimal multi-cue strategy during testing achieved significantly higher accuracy than those identified as having used suboptimal, single feature or random pattern strategies. This is consistent with the findings of Gluck et al. (2002) in which multi-cue users generated a higher proportion of optimal responses than one-cue users during the WPT, as well as the findings of Vallila-Rohter and Kiran (2015) in which optimal strategy users performed significantly better than single feature or random pattern strategy users during the same task that was used in this study. This study’s findings add evidence that strategy use is consequential for outcomes and further solidifies confidence in modeled strategies. Overall, more participants developed an optimal strategy under feedback-based training than observational training, and those who used an optimal strategy outperformed those who used suboptimal strategies.

The researchers had hypothesized that participants would achieve higher testing accuracy under feedback training. While a greater number of participants developed optimal multi-cue strategies during feedback learning, this did not translate to significant differences in accuracy between observational and feedback-based conditions. Stimuli in the current task had more dimensions (10 feature dimensions) compared with two to four feature dimensions utilized in other studies (Rustemeier et al., 2013; Marchant and Chaigneau, 2021), which may have contributed to the nonsignificant differences in accuracy between observational and feedback training conditions. In addition, the probabilistic nature of the task makes it such that features are associated more frequently with one category or another, making it possible to achieve high accuracies even when utilizing single feature strategies. Sample size could also be a factor.

Consistent with expectations and prior literature, FRN amplitudes following negative feedback were larger than FRN amplitudes following positive feedback – a common characteristic of the FRN (Holroyd and Coles, 2002; Ernst and Steinhauser, 2012). This finding increases confidence that the FRN was successfully isolated in this study. Contrary to expectations, larger P3a amplitudes to negative feedback than positive feedback were not observed. This may be related to the fact that feedback in a probabilistic task with multidimensional stimuli is not deterministic on a trial by trial basis. Instead, feedback provides the learner with information about the accuracy of a single *response* but does not provide the learner with information about which stimulus characteristics they classified correctly/incorrectly in that single trial, meriting a learner’s attention to both positive and negative feedback. It is also worth noting that evidence for both the presence and direction of a valence effect on P3a amplitude is varied (Yeung and Sanfey, 2004; Sato et al., 2005; Wu and Zhou, 2009). While this study’s finding of a nonsignificant valence effect on P3a amplitude is in contrast to the findings of Rustemeier et al. (2013) who similarly measured P3a amplitude during a probabilistic learning task, evidence of a valence effect on P3a amplitude remains mixed.

Contrary to hypotheses that predicted larger P3a in single feature strategy users early in training, and larger FRN amplitudes in multi-cue users, single feature strategy users were the only ones to show significant ERP effects, demonstrating a significant decrease in FRN amplitude from early to late training. The single feature strategy users’ significant decrease in FRN amplitude from early to late training aligns well with the utility account of the FRN, which suggests that a decreasing FRN over time reflects that the utility of feedback decreases over time (Arbel et al., 2014). In this case for the single feature strategy users, a decrease in FRN amplitude in late training may reflect their reduced use of feedback to inform their responses, as they continued to base category responses on a single feature despite receiving negative feedback. For example, single feature strategy users who based their responses on body pattern (stripes or spots) consistently categorized stimuli with stripes in Category A despite receiving negative feedback on a proportion of trials. The lack of strategy adaptation in the face of negative feedback suggests a decreased dependence or utilization of feedback, whether conscious or unconscious. The single feature strategy users’ significant decrease in FRN amplitude from early to late training may also be interpreted in relation to the expectancy account of the FRN (Ferdinand et al., 2012), reflecting a reduced prediction error as described in the prediction of response–outcome (PRO) theory (Alexander and Brown, 2010, 2011). Within this framework, the case of a decrease in FRN amplitude in late training among single feature strategy users, irrespective of feedback valence, may be due to the increased predictability of feedback over the course of the task. The current study’s findings also differ from Rustemeier et al. (2013) who observed significant group differences in P3a amplitude but nonsignificant group differences in FRN amplitude. Rustemeier et al. (2013) suggested that incorporating a mixed strategy might have led to significant findings with FRN amplitude. The current study incorporated a random strategy intended to reduce the number of falsely identified multi-cue and single feature strategy fits, and may have led to a sharper distinction of single feature and multi-cue strategy users that led FRN differences to be detected even in this small pilot sample of participants.

In contrast, multi-cue and random pattern strategy users did not show a significant change in FRN amplitude from early to late training, suggesting continued use of feedback. Similar studies to the current study have examined the FRN in the context of learning where feedback is more informative. The current study, as well as the study by Rustemeier et al. (2013), uniquely examined the FRN in the context of a probabilistic task, where information must be accrued over multiple trials and dimensions. Feedback provided during a probabilistic learning task is informative but not deterministic on a trial by trial basis. Since information must be accrued over multiple trials and dimensions, it is likely that the multi-cue and random strategy users utilized feedback throughout the course of learning as learners update a nuanced, multi-dimensional category representation.

The current study found no significant main effects or interaction effects related to the P3a. Therefore, a similar relationship between strategy and P3a amplitude as found by Rustemeier et al. (2013) was not observed. This may be related to the fact that that the stimuli in this study’s task contained 10 features/dimensions, whereas the stimuli used in the WPT by Rustemeier et al. (2013) contained four dimensions. With a greater number of stimulus dimensions, it may have been more challenging for participants to evaluate negative feedback following a single trial in order to correct their performance in subsequent trials, as each selection/trial involved a greater number and/or more complex combinations of dimensions to consider and evaluate.

There are several potential limitations to note. First, the researchers had all participants complete the observational condition first and the feedback-based condition second in order to keep the observational condition free of any outcome prediction that might result from exposure to the feedback-based (outcome generating) condition. Future iterations of this work should consider counterbalancing task order to see if the feedback-based condition still results in a greater number of multi-cue strategy users. Second, while as few as 6–10 trials per condition can be adequate to detect significant ERP effects (Boudewyn et al., 2018), future work should incorporate a greater number of training trials. Future directions for this work may include: collecting standardized cognitive data from participants in order to draw further conclusions about the individual learners, and to further relate learning, strategy, and feedback analyses to standardized cognitive metrics; collecting qualitative data from participants (e.g., asking them about what approach they took during the training and testing phases and/or which characteristics they think constitute each category); and increasing the study sample size, since stratifying participants into strategy/trajectory groups reduces the number of individuals within each group used in statistical comparisons.

## Conclusion

In conclusion, the findings of this study provide further evidence that feedback-based training may be more likely to result in optimal strategy development for probabilistic, complex category learning. Furthermore, those who developed an optimal strategy outperformed suboptimal strategy users, and thus, feedback-based training may also result in greater learning accuracy for probabilistic, complex category learning. Few studies have combined strategy analyses with measures of feedback processing. This study’s findings suggest that comparing measures of feedback processing between early and late-phase training of probabilistic, complex category learning tasks may reflect processing and utilization of feedback and may further illuminate differences among individual learners. However, future studies that implement probabilistic tasks involving stimuli with a greater number of dimensions, such as the ones used in this study, may decide to lengthen their training phases in order to allow for higher learning accuracies, which may assist in their ability to draw group-level and individual-level differences in learning.

## Data Availability Statement

The raw data supporting the conclusions of this article will be made available by the authors, without undue reservation.

## Ethics Statement

The studies involving human participants were reviewed and approved by Mass General Brigham Institutional Review Board. The Participants provided their written informed consent to participate in this study.

## Author Contributions

SV-R and YA contributed to conception and design of the study and wrote sections of the manuscript. VT-B performed the statistical analysis and wrote the first draft of the manuscript. All authors interpreted findings together. All authors contributed to the article and approved the submitted version.

## Funding

This work was supported by the National Institute on Deafness and Other Communication Disorders (NIDCD) of the National Institutes of Health under award number R21DC019203, and funds from the Christopher Norman Educational Fund at the MGH Institute of Health Professions. The content is solely the responsibility of the authors and does not necessarily represent the official views of the National Institutes of Health.

## Conflict of Interest

The authors declare that the research was conducted in the absence of any commercial or financial relationships that could be construed as a potential conflict of interest.

## Publisher’s Note

All claims expressed in this article are solely those of the authors and do not necessarily represent those of their affiliated organizations, or those of the publisher, the editors and the reviewers. Any product that may be evaluated in this article, or claim that may be made by its manufacturer, is not guaranteed or endorsed by the publisher.

## Footnotes

^1^ www.pstnet.com
